# Choledochal Malformation in Children: Lessons Learned from a Dutch National Study

**DOI:** 10.1007/s00268-017-4064-x

**Published:** 2017-06-06

**Authors:** Nederlandse Studiegroep voor Choledochus Cysten/malformaties (NeSCHoC), Maria. H. A. van den Eijnden, Ruben H. J. de Kleine, Ivo de Blaauw, Paul G. J. M. Peeters, Bart P. G. Koot, Matthijs W. N. Oomen, Cornelius E. J. Sloots, W. G. van Gemert, David C. van der Zee, L. W. E. van Heurn, Henkjan J. Verkade, Jim C. H. Wilde, Jan B. F. Hulscher

**Affiliations:** 1 Department of Pediatric Surgery, University of Groningen, University Medical Center Groningen, Hanzeplein 1, HPC BA20 Postbus 30.001, 9700RB Groningen, The Netherlands; 2 Department of Hepato-Pancreatico-Biliary Surgery and Liver Transplantation, University of Groningen, University Medical Center Groningen, Hanzeplein 1, 9700RB Groningen, The Netherlands; 3 Surgery-Division of Pediatric Surgery, Amalia Children’s Hospital, Radboud University Nijmegen, Medical Center, Geert Grooteplein 10, 6525GA Nijmegen, The Netherlands; 4 Department of Pediatric Surgery, Emma Children’s Hospital, Academic Medical Center, University of Amsterdam, Meibergdreef 9, 1105AZ Amsterdam, The Netherlands; 5 Department of Pediatric Surgery, Sophia Children’s Hospital, Rotterdam, Erasmus Medical Center, Erasmas University, ‘s-Gravendijkwal 230, 3015CE Rotterdam, The Netherlands; 6 Department of Pediatric Surgery, University Medical Center Maastricht, University of Maastricht, P. Debyelaan 25, 6229HX Maastricht, The Netherlands; 7 Department of Pediatric Surgery, University Medical Center Utrecht, University of Utrecht, Heidelberglaan 100, 3584CX Utrecht, The Netherlands; 8 University Center of Pediatric Surgery of Western Switzerland, Division of Pediatric Surgery, University Hospitals of Geneva, Rue Gabrielle-Perret-Gentil 4, Geneva, 1205 Switzerland

## Abstract

**Introduction:**

A choledochal malformation (CM) is a rare entity, especially in the Western world. We aimed to determine the incidence of CM in the Netherlands and the outcome of surgery for CM in childhood.

**Methods:**

All pediatric patients who underwent a surgical procedure for type I–IV CM between 1989 and 2014 were entered into the Netherlands Study group on choledochal cyst/malformation. Patients with type V CM were excluded from the present analysis. Symptoms, surgical details, short-term (<30 days) and long-term (>30 days) complications were studied retrospectively.

**Results:**

Between January 1989 and December 2014, 91 pediatric patients underwent surgery for CM at a median age of 2.1 years (0.0–17.7 years). All patients underwent resection of the extrahepatic biliary tree with restoration of the continuity via Roux-en-Y hepaticojejunostomy. Twelve patients (12%) were operated laparoscopically. Short-term complications, mainly biliary leakage and cholangitis, occurred in 20 patients (22%), without significant correlations with weight or age at surgery or surgical approach. Long-term postoperative complications were mainly cholangitis (13%) and anastomotic stricture (4%). Eight patients (9%) required radiological intervention or additional surgery. Surgery before 1 year of age (OR 9.3) and laparoscopic surgery (OR 4.4) were associated with more postoperative long-term complications. We did not observe biliary malignancies during treatment or follow-up.

**Conclusion:**

Surgery for CM carries a significant short- and long-term morbidity. Given the low incidence, we would suggest that (laparoscopic) hepatobiliary surgery for CM should be performed in specialized pediatric surgical centers with a wide experience in laparoscopy and hepatobiliary surgery.

## Introduction

A choledochal malformation (CM) is a rare biliary entity with an estimated incidence of 1:100–150,000 live births in Western countries [1, 2]. In the Asian population, the incidence can be as high as 1:1000 live births. The reason for this Asian preponderance is still unknown [3]. CM is primarily a childhood disease: Up to 80% of patients are diagnosed before 10 years of age [4–6].

Mainstay of treatment is resection of the affected (extrahepatic) bile ducts with subsequent restoration of the continuity between biliary tract and intestines [4, 5, 7, 8]. This complex surgery is associated with significant short- and long-term morbidity [8–10]. Timing of surgery, especially in asymptomatic infants, is difficult. Early surgery might prevent CM-related symptoms, while later surgery might diminish surgery-related complications [11–13]. In the Netherlands, most resections for CM are performed via a laparotomy [14]. However, the laparoscopic approach is gaining popularity [14–17].

Most reports on CM are case series from a single institution. In the present study, we describe results from over 25 years of experience with CM in the Netherlands. To this end, we collected data from the six Dutch pediatric surgical centers. We identified short- and long-term postoperative complications and searched for possible risk factors associated with worse postoperative outcomes. This way we aimed to identify possible targets for improvement of care. Our main focus was on differences in outcomes between early surgery versus delayed surgery and the differences between laparoscopic and open resection.

## Patients and methods

We included all Dutch pediatric patients who were operated for CM < 18 years of age in the Netherlands between January 1, 1989 and December 31, 2014. Patients who were diagnosed with a type V CM were excluded. Data were collected from all academic pediatric surgical centers performing surgery for CM, resulting in a national registry: “Nederlandse Studiegroep voor CHoledochus Cysten/malformaties (NeSCHoC).”

Patient charts were reviewed retrospectively. The primary investigator (ME) collected data concerning patient demographics, age at diagnosis, type of CM according to Todani [18], age at surgical intervention, type of surgical intervention, surgical data and postoperative complications. Postoperative complications were defined as short-term (<30 days) and long-term (>30 days) complications following the Clavien–Dindo classification [19]. Postoperative complications were defined as follows: cholangitis as fever (>38.5) with abdominal pain and jaundice, pancreatitis as abdominal pain with elevated serum amylase, wound infection as red discoloration of the surgical and/or drain wound, bile leakage as abdominal complaints and proven bilirubin in the abdominal fluid, intra-abdominal infections as fever and a proven bacterial infection confirmed via culture, ileus as >1 day of abdominal distension, vomiting and the absence of defecation and gas, liver(bile)stones as intrahepatic bile stones confirmed by ultrasonography, liver abscesses as circumscribed infection in the liver confirmed by ultrasonography and liver fibrosis/cirrhosis as confirmed histologically via liver biopsy.

We also collected the occurrence of biliary malignancies and survival data. We compared the outcomes after laparoscopic resection with the outcomes after resection via laparotomy, and the outcomes after surgery before 1 year of age with outcomes after surgery after 1 year of age. Follow-up was performed using the hospital databases. As surgical treatment of CM is only performed in a pediatric surgical center, all postoperative complications were collected from the patients’ electronic or paper files in the pediatric surgical center where the resection was performed. Follow-up analysis was conducted from January 1, 1989 until of October 1, 2015. The study protocol was approved by the University Medical Center Groningen Ethics Committee (METC2015/115).

### Statistical analysis

Differences between groups were tested with the *t* test, Mann–Whitney U and Wilcoxon signed rank test as appropriate. To determine statistical differences between ordinal values, the Chi-square or Fishers’ exact test was used as appropriate, and odds ratio were calculated. Potential prognostic factors for overall postoperative complication-free survival were evaluated using univariate analysis. We used the log-rank test to compare groups for postoperative complication-free survival after surgery. *p* values < 0.05 were considered statistically significant. The SPSS IBM 22 package (Armonk, New York, United States) was used for statistical analysis.

## Results

We identified 91 patients <18 years who underwent surgical intervention for CM type I–IV in the Netherlands between January 1, 1989 and December 31, 2014. Based on this data the Dutch specimen-proven incidence of I–IV CM in patients <18 years is at least 1:59.000 live births [20].

### Patient cohort

Median age at the start of CM-related symptoms was 1.0 years (1 day—16.4 years); median age at diagnosis was 1.9 years (1 day—17.1 years). There were 66 type I (73%), two type II (2%), no type III, 17 type IVa (19%) and one type IVb (1%) according to the Todani classification [18]. In five patients (5%), the Todani classification could not be retrieved. There were 27 males (30%) and 64 females (70%), leading to a male–female ratio of 1:2,4. Comorbidity was present in three patients and consisted of Li-Fraumeni P53 mutation, congenital disorder of the glycosylation type Ia and galactosemia.

### Surgery

Surgical parameters are depicted in Table 1. All patients underwent resection of the extrahepatic biliary tree with Roux-en-Y hepaticojejunostomy. Parenchyma resection during primary surgery was not performed. Seventy-nine patients (87%) were operated via laparotomy and 12 patients (13%) via laparoscopy. In one patient, laparoscopy was converted to laparotomy. Hospital stay was significantly shorter after laparoscopic resection (5 vs 9 days, *p* = 0.02) and in patients >1 year (8 vs 12 days, *p* < 0.01). Operative time was significantly shorter in patients treated via laparotomy (3.5 vs 5.5 h, *p* = 0.02).

**Table 1:** Surgical parameters in patients undergoing surgery for choledochal malformation in the Netherlands between January 1, 1989 and December 31, 2014

	All (*n* = 91)	Laparoscopy (*n* = 12)	Laparotomy (*n* = 79)	Surgery <1 year of age (*n* = 30)	Surgery >1 year of age (*n* = 61)
Median age at surgery (years)	2.1 (0.03–17.7)	1.1 (0.03–8.9)	2.3 (0.06–17.7) (0.2)	0.2 (0.03–1.0)	4.3 (1.1–17.7)
Median weight at surgery (kg)	10.6 (2.8–52.5) (*n* = 66)	9.0 (2.8–28) (*n* = 11)	11.0 (3.7–52.5) (*n* = 55) (*p* = 0.3)	5.2 (2.8–12) (*n* = 24)	15.8 (9–52.5) (*n* = 42)
Median operative time (hrs)	3.5 (1.2–9.3) (*n* = 81)	5.5 (2.0–7.2) (*n* = 11)	3.5 (1.2–9.3) (*n* = 70) (*p* = 0.03)	3.5 (1.5–6.3) (*n* = 27)	3.5 (1.7–9.3) (*n* = 54) (*p* = 0.2)
Median hospital stay (days)	9 (2–61) (*n* = 88)	5 (4–16)	9 (2–61) (*n* = 76) (*p* = 0.02)	12 (4–61) (*n* = 29)	8 (2–29) (*n* = 59) (*p* < 0.01)

*kg* kilograms, *hrs* hours

### Postoperative course

Short-term complications occurred in 20 patients (22%). Eight patients (9%) required reoperation and/or ICU admittance for short-term complications. One patient died within 30 days postoperatively due to an abdominal compartment syndrome in combination with congenital disorder of the glycosylation type Ia. Therefore, analysis of long-term complications was performed in 90/91 patients.

Median follow-up was 13.6 years (0.8–26.0). Long-term complications occurred in 16 patients (16/90, 17%). Eight patients (8/90, 9%) required reoperation or radiological intervention. One patient (1/90, 1%) died >30 days postoperatively because of non-related disease.

### Risk factors for postoperative complications

#### Surgical technique

Tables 2 and 3 depict short-term (<30 days) and long-term (>0 days) postoperative complications, respectively. Two patients (2/12, 18%) developed short-term complications after laparoscopic surgery versus 18 patients (18/79, 24%) after laparotomy (*p* = 0.5). Five patients (5/12, 42%) developed long-term complications after laparoscopic surgery versus 11 patients (11/78, 14%) after laparotomy (*p* = 0.04). The majority of long-term complications occurred within 2 years after surgery (65%). Complications consisted mainly of cholangitis (*n* = 12) and anastomotic strictures (*n* = 4).

**Table 2:** Short-term complications in patients after resection of choledochal malformation

	All (*n* = 91)	Laparoscopy (*n* = 12)	Laparotomy (*n* = 79)	Surgery <1 year of age (*n* = 30)	Surgery >1 year of age (*n* = 61)
Total number of patients	20 (22%)	2 (17%)	18 (23%) (*p* = 1.0)	10 (33%)	10 (16%) (*p* = 0.1)
CD I					
Total number of patients	4 (4%)	0 (0%)	4 (5%)	3 (10%)	1 (2%)
Anuria	1	0	1	1	0
Unexplained fever	1	0	1	1	0
Ascites	1	0	1	1	0
Pancreatitis	1	0	1	0	1
CD II					
Total number of patients	8 (9%)	1 (8%)	7 (9%)	4 (13%)	4 (7%)
Unexplained fever	1	0	1	1	0
Cholangitis	3	1	2	3	0
Impetigo	1	0	1	0	1
Skin infection	2	0	2	0	2
Bile leakage	1	0	1	0	1
CD III					
Total number of patients	6 (8%)	1 (8%)	5 (6%)	2 (7%)	4 (7%)
Bile leakage	4	0	4	1	3
Deep infection	2	0	2	0	2
Pleural fluid	1	0	1	0	1
Ileus	1	0	1	0	1
Duodenum perforation	1	1	0	1	0
CD IV					
Total number of patients	1 (1%)	0	1 (1%)	1 (3%)	0 (0%)
Bile leakage	1	0	1	1	0
Liver abscesses	1	0	1	1	0
CD V					
Total number of patients	1 (1%)	0	1 (1%)	0	1 (2%)
Abdominal compartment syndrome with CDG type Ia comorbidity	1	0	1	0	1

This table describes the total number of complications that occurred and type of complications; some patients encountered more than one short-term complications

*CD* Clavien–Dindo, *CDG* congenital disorder of the glycosylation

**Table 3:** Long-term complications in patients after resection of choledochal malformation

	All (*n* = 90)	Laparoscopy (*n* = 12)	Laparotomy (*n* = 78)	Surgical intervention <1 year of age (*n* = 30)	Surgical intervention >1 year of age (*n* = 60)
Total	16 (18%)	5 (42%)	11 (14%) (*p* = 0.03)	12 (40%)	4 (7%) (*p* < 0.01)
Cholangitis	12 (13%)	4 (33%)	8 (10%)	9 (30%)	3 (5%)
Stricture	4 (4%)	2 (17%)	2 (3%)	3 (10%)	1 (2%)
Cirrhosis	1 (1%)	0	1 (1%)	1 (3%)	0
Mechanical ileus	1 (1%)	0	1 (1%)	1 (3%)	0
Gallstones	2 (2%)	0	2 (3%)	1 (3%)	1 (2%)
Abscesses	2 (2%)	0	1 (1%)	1 (3%)	0 (0%)
Portal hypertension	2 (2%)	0	2 (3%)	2 (7%)	0 (0%)
Incisional hernia	1 (1%)	0	1 (1%)	0	1 (2%)
Secondary parenchyma resection	1 (1%)	0	1 (1%)	1 (3%)	0
Incomplete CM resection	1 (1%)	1 (8%)	0	0	1 (2%)
Complications requiring radiological intervention or re-surgery	8 (9%)	3 (25%)	5 (6%) (*p* = 0.07)	6 (20%)	2 (3%) (*p* = 0.02)

This table describes the total number of complications that occurred and type of complications; some patients encountered more than one long-term complications

*CM* choledochal malformation

#### Age at the time of surgical intervention

As shown in Tables 2 and 3, 10 patients (10/30, 33%) who underwent surgery before 1 year of age developed short-term complications versus 10 patients (10/61, 18%) who underwent surgery after 1 year of age (*p* = 0.2). There were significantly more long-term complications in patients operated before 1 year of age (12/30, 39%) when compared with patients operated after 1 year of age (4/60, 8%, *p* < 0.01).

#### Complication-free survival in relation to type of surgery and age at surgery

Median complication-free survival was 1.6 years (0.2–8.7 years) after laparoscopic surgery and 13.8 years (0.1–25.9 years) after laparotomy (*p* < 0.01). There was no difference in median complication-free survival between patients undergoing surgery before 1 year of age and patients undergoing surgery after 1 year of age.

### Risk factors for postoperative short- and long-term complications

To identify risk factors for short- and long-term complications, several patient and surgery-related parameters were tested (Table 4). There were no significant risk factors for the occurrence of short-term postoperative complications. However, complications were more prevalent in patients operated before 1 year of age than in patients operated at an older age (after 2 years). Surgical intervention before 1 year of age and laparoscopic resection were associated with a significantly higher risk for long-term complications (OR 4.4 and 9.3, respectively).

**Table 4:** Risk factors for short- and long-term complications (univariately)

	Odds ratio	95% confidence interval
Short-term complications		
Surgery <1 year of age	2.5	0.8–7.1
Long-term complications		
Surgery <1 year of age	9.3	2.7–32.5
Laparoscopic technique	4.4	1.2–16.2
Occurrence of a short-term complication	1.2	0.4–4.3

## Discussion

In the present series we describe results from the first nationwide database from a Western country; consisting of 91 patients younger than 18 years who underwent surgery for choledochal malformation in the Netherlands. The incidence of CM was much higher than expected. Postsurgical morbidity was significant, about half of the patients who developed a complication needed radiological, endoscopical or surgical re-intervention. In univariate analysis, surgery within the first year of life and laparoscopic approach were associated with long-term complications. A multivariate analysis is necessary to investigate whether both age at surgery and laparoscopic resection are independently associated with a higher incidence in complications. The low incidence of the condition prevented multivariate analyses, despite the national collection of data over a prolonged period.

We found a specimen-proven surgical incidence of 1:59,000 live births in the present series. This incidence is at least two to three times higher than expected (1:100–150,000) for non-Asian countries [1, 2]. The current incidence might even be an underestimation: We expect that the incidence will probably even be higher when a similar national registry for patients >18 years of age is established. We might also not have been able to identify all children, as children might have been operated in another country or not operated at all.

Short-term postoperative complications are poorly reported in the literature, but our present data seem similar to the available data with a substantial overall rate of 22% [9, 21–23]. The long-term complication rate in the literature ranges between 5 and 15%, and reoperation rates ranges between 1 and 20% [9, 21–25]. Cholangitis occurs in 1–9% of the patients, stricture of the biliodigestive anastomosis occurs in 1–9% of the patients, and there is a reoperation rate of 1–20% [9, 21–25]. We believe these numbers show a wide distribution mainly due to the variable length of follow-up [9, 21–25]. The present series demonstrates a relatively high overall long-term complication rate of 18%. There seems a clear decrease in complication rate with an increase in age. Technically, surgery in older children is easier when compared to young children. This especially holds true for the biliodigestive anastomosis [11–13]. The difficulties in hepatobiliary surgery in young children have also been described in patients undergoing liver transplantation [26]. It is tempting to speculate that complication rates could therefore also be decreased by avoiding surgery in asymptomatic patients who have not reached 1 year of age. This might also be the case for anesthesiologic effects, which decrease sharply with age in children [27, 28]. As surgery for CM in the asymptomatic patient can be considered prophylactic surgery, the risks of such major undertaking have to be weighed against the risks of watchful waiting. In asymptomatic patients, careful monitoring with regular ultrasound and laboratory checks until the child has reached 1 year of age (corresponding to ca. 10 kg) should be able to early identify patients at a high risk for progressing to CM-related complications which should therefore be operated early. This way, one should be able to prevent technically more demanding surgery after inflammation of the cyst. In the presence of CM-related complications, patients should be operated as soon as their clinical condition permits.

In the present series, 42% of the patients undergoing laparoscopic surgery suffered from a long-term complication. Children undergoing laparoscopic resection have a tendency to be younger than patients undergoing laparotomy (1.1 vs 2.3 years, *p* = 0.2). This may have influenced the results, as surgery in younger children is associated with a higher incidence of complications in our series. Moreover, there can be a significant selection bias because the patients had not been randomized for laparoscopic or open surgery. These data suggest that we still might be in our learning curve. Laparoscopic (hepatobiliary) pediatric surgery has a long learning curve, especially in young children [29–31]. From the results of this study, no definitive conclusions can therefore be drawn regarding the laparoscopic approach of CM. While we do not advocate the avoidance of laparoscopic resection, we do argue for centralization of these procedures to increase numbers and thereby shorten the learning curve. Davenport et al. [32] demonstrated that centralization of care led to improvement of the outcomes of biliary atresia, another rare pediatric hepatobiliary disease. Centralization of care for children with CM may also lead to better outcomes.

A potential long-term consequence of CM is the development of a malignancy. We did not observe any malignancies during follow-up, but we do realize that our cohort is still relatively young. In order to detect malignancies in the Western CM patients, a longer follow-up has to be established. Lifelong follow-up therefore remains important. Several other studies investigated the malignancy risk after excision of a CM [22, 29, 33, 34]. The malignancy risk is considered to increase with age at surgery, and the cumulative biliary malignancy risk 25 years after primary surgery has been reported to be as high as 11% [30]. These data stem from the high volume Asian centers and could be different in patients in Western countries. In a recent large cohort, Soares et al. [22] mentioned the development of malignancy in 0.7% in Western patients. However, with a median follow-up of 2.7 years in their pediatric cohort and 11 years in our cohort (with no malignancies), the malignancy risk of Western CM patients operated at infancy seems low.

Theoretically, the retrospective nature of our study could have underestimated the incidence of postoperative complications. In the Netherlands, treatment of CM is only performed in one of the six pediatric surgical centers. Therefore, most (if not all) postoperative complications are expected to be registered in the patients’ files. The NeSCHoC is the first nationwide database on choledochal malformations spanning several decades. This enabled us to thoroughly investigate a rare disease. This study is one of the first to fully describe the surgical outcomes in patients diagnosed and operated on CM <18 years of age. The retrospective nature of the study has led to an unavoidable gap in data in several areas, not to mention the changes in care that have taken place over 25 years. However, we feel that it is a major step forward to address relevant issues of this rare condition. NeSCHoC is now converted into a prospective database. The present database still contains a relatively small cohort of patients precluding multivariate analysis and thereby further detailed analysis of risk factors. Such analyses should be done when larger numbers become available, for which international cooperation (for instance, via an online registry such as the Biliary Atresia and Related Disease (BARD) database) is desirable.

## Conclusion

While CM remains a rare disease, the incidence of CM in the Netherlands is much higher than expected. Surgery for CM carries significant short-term and long-term morbidity warranting a lifelong follow-up. In the present series, a younger age at surgery and laparoscopic surgery were associated with a higher risk of complications. These findings suggest that postponing surgery in asymptomatic children can decrease complication rates. Since laparoscopic surgery was often performed in the younger age group, it is hard to determine whether age and laparoscopic surgery are independently associated with a higher number of complications. Laparoscopic hepatobiliary surgery in small children should only be performed by surgeons with a wide experience in laparoscopy and hepatobiliary surgery. We feel that in the Dutch situation this can only be reached via centralization of care of this rare disease.
